# NGAL Usefulness in the Intensive Care Unit Three Hours after Cardiac Surgery

**DOI:** 10.5402/2013/865164

**Published:** 2012-11-27

**Authors:** Geoffray Delcroix, Nicole Gillain, Martial Moonen, Luc Radermacher, François Damas, Jean-Marc Minon, Vincent Fraipont

**Affiliations:** ^1^Department of Intensive Care, CHR Citadelle, Boulevard du 12ème de ligne 1, 4000 Liège, Belgium; ^2^Department of Laboratory Medicine, CHR Citadelle, Boulevard du 12ème de ligne 1, 4000 Liège, Belgium; ^3^Department of Nephrology, CHR Citadelle, Boulevard du 12ème de ligne 1, 4000 Liège, Belgium

## Abstract

*Objective*. Neutrophil gelatinase-associated lipocalin (NGAL) measured by a research ELISA is described as an early marker of acute kidney injury (AKI). The aim of this study is to define the usefulness of plasma NGAL (pNGAL) and urine NGAL (uNGAL) measured with platform analysers to detect AKI 3 hours after cardiac surgery in fifty adult patients. *Methods and Main Results*. pNGAL and uNGAL were measured before and 3 hours after cardiac surgery. AKI, defined following the acute kidney injury network definition, was observed in 17 patients. pNGAL was >149 ng/mL in 8 patients with AKI, two of them died in the follow-up. We also observed elevated pNGAL in 8 patients without AKI. Only one uNGAL was >132 ng/mL among the 15 AKI patients. Sensitivity of pNGAL for prediction of AKI is 47% and specificity is 75.7%. The positive likelihood ratio (LR+) is 1.9 and negative likelihood ratio (LR−) is 0.7. uNGAL performance is slightly improved when reported to urinary creatinine. Following this study, a ratio >62 ng/mg assure a sensitivity of 66.6% and a specificity of 78.5%. LR+ is 3 and a LR− is, 0.42. *Conclusions*. Three hours after cardiac surgery, pNGAL predicts AKI with a low sensitivity and specificity.

## 1. Introduction

Acute kidney injury (AKI) is a major postoperative complication after cardiac surgery [1, 2]. This is associated with increased mortality, prolonged ICU length of stay, and sometimes prolonged kidney dysfunction [3]. Prompt diagnosis could lead to hemodynamic optimization and could prevent progression of AKI.

The most recognised criteria for the diagnosis of AKI are currently based on the RIFLE score or AKIN modified score which is based on the serum creatinine variation and the urine output [4, 5]. It is commonly described that serum creatinine elevation is a late indicator of kidney dysfunction and that the steady state is reached lately (sometimes up to 48–72 hours) while half the kidney function was already lost [6, 7].

Last few years, some new biomarkers appear to be promising for the rapid diagnosis of AKI compared to classical indicators such as serum creatinine or urine output: cystatin C, interleukin 18 (IL-18), kidney injury molecule (KIM1), liver fatty acid binding protein (LFABP), and neutrophil gelatinase-associated lipocalin (NGAL). 

NGAL appears to be the most promising marker, but there are conflicting observations concerning the validity of this expensive test.

Neutrophil gelatinase-associated lipocalin (NGAL) is a member of the lipocalin superfamily of more than 20 structurlaly related secreted proteins and thought to participate in ligand transport with a *β*-barreled calyx. Human NGAL was originally isolated in 2003 by Mishra et al. as a 25 kDa protein covalently bound to gelatinase from neutrophils [8]. This small protein is resistant to proteinase and rapidly upregulated and expressed in response to ischemic or nephrotoxic kidney injury [8, 9]. These properties of NGAL explain the increasing enthusiasm to establish it as an ideal and early biomarker of AKI [10].

The purpose of this study is to evaluate the efficiency of a single urine and plasmatic NGAL dosage to detect AKI earlier than traditional indicators, after adult cardiac surgery, with standardized techniques of measurement.

## 2. Materials and Methods 

This observational cohort study takes place in the general intensive care unit of a tertiary general hospital of 1000 beds. Ethical approval was obtained from the local Ethical Committee of the CHR Citadelle.

Fifty adult cardiac surgical patients were successively enrolled in this study from 12 January to 15 March 2010.

pNGAL was performed before the surgery among 32 of them, and pNGAL was performed for all 50 patients three hours after surgery. uNGAL was performed in 43 of them 3 hours after surgery. 

Surgery consisted in myocardial revascularization (*n* = 37) or valvuloplasty (*n* = 8) or combined (*n* = 4) and one myxoma resection plus valvuloplasty under cardiopulmonary bypass.

The glomerular filtration rate (GFR) was calculated with CKD-EPI equation [11]. This equation is based on plasma creatinine levels the day before the surgery which were determined with a Jaffé compensated kinetic method adapted for a modular (Roche) autoanalyser. The plasma NGAL (pNGAL) was measured with Triage kit for point of care (Alere-Biosite) on EDTA sample collected for haematology the day before the surgery and 3 hours after the return of the operating room. The urinary NGAL (uNGAL) was measured with the ARCHITECT analyzer (Abbott Diagnostics) on urine sample especially collected for the study from indwelling bladder catheter at the same time among 43 of the 50 patients. Plasmatic creatinine was regularly measured after the surgery (3, 6, and 12 hours after surgery and every 12 hours for the following 48 hours). Cut-offs for the pNGAL and uNGAL were >149 ng/mL and >130 ng/mL, respectively. These expected ranges reported by the manufacturer are the values of the 95th percentile of a population without renal deficiency. Urine creatinine was measured on urine sample on modular (Roche) autoanalyser and uNAGL ratio to creatinine calculated for urine volume correction. Any reference range was defined by Abbott for this ratio.

Physicians were blinded of pNGAL and uNGAL results and laboratory staff of the clinical outcome. The sensitivity, specificity, likelihood ratio, and ROC curve were calculated with GraphPad Prism software.

## 3. Results

Fifty patients, 12 women and 38 men aged from 31 to 84 years, were included (mean 67.7 ± 9.98).

The glomerular filtration rate (GFR) before surgery of the 50 patients, calculated from plasma creatinine and CKD-EPI equation, was >60 mL/min for 42 patients, between 30–60 mL/min for 6 and <30 mL/min for 2 patients. pNGAL performed before the surgery in 32 of them was <149 ng/mL. Seventeen patients (34%) developed AKI defined following the acute kidney injury network: 9 patients stage 1 (increase of creatinine ≥0.3 mg/dL or ≥1.5–2 fold the baseline), 2 patients stage 2 (increase ≥2–2.9 fold the baseline), and 6 patients stages 3 (increase ≥3 fold the baseline or creatinine ≥ 4 mg/dL after a rise of 4.97 mg/dL or treatment with renal replacement therapy). Two patients of this stage 3 group died from sepsis and multiorgan failure.

The individual values of the patients who developed AKI are reported in Table 1 according to the timing of the creatinine rise and the stage of the AKI.

pNGAL was >149 ng/mL (156–301 ng/mL) among 8 patients with AKI, two of them being the deceased patients. (pNGAL 301 ng/mL uNGAL 4.2 ng/mL for one; pNGAL 161 ng/mL uNGAL 37 ng/mL for the other). 

We observed also elevated pNGAL (161–239 ng/mL) among 8 patients without AKI. 

**Figure 1:**
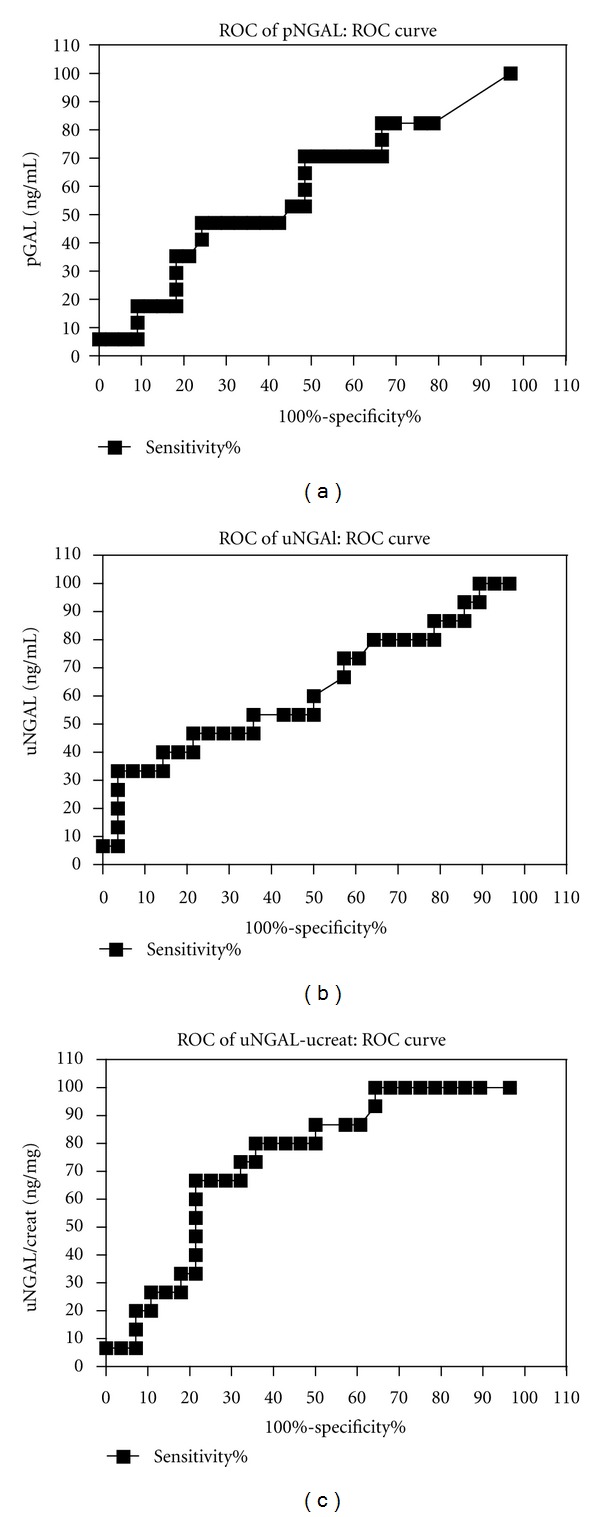
AUC-ROC for pNGAL, uNGAL, and uNGAL/ucreat ratio.

The sensitivity and the specificity of the pNGAL for the prediction of AKI in this study are of 47% and 75.7% respectively. The positive likelihood ratio (LR+) was 1.9, and the negative likelihood ratio (LR−) was 0.7. The AUC-ROC for pNGAL is 0.58 (95%CI 0.41–0.753; *P* = 0.33) (Figure 1). 

Among the 15 AKI patients for which urine was collected, only one uNGAL was >130 ng/mL (166 ng/mL).

The sensibility for AKI detection of uNGAL is 6.67% with a specificity of 96.4%. The AUC-ROC was 0.62 (95%CI 0.44–0.809; *P* = 0.18) (Figure 1). 

The uNGAL/creatinine expressed in ng/mg varied from 9 to 320 in non-AKI patients and from 24 to 506 in AKI patients. The ROC curve demonstrated that a 62 ng/mg ratio provides a sensibility of 66.6% and a specificity of 78.5%. The AUC-ROC was 0.62 (95%CI 0.44–0.809; *P* = 0.18) for this ratio. Using the ratio uNGAL to the urinary creatinine improved sensitivity to 66.6% and specificity to 78.5% (Table 2). The AUC-ROC for this one is 0.73 (95%CI 0.58–0.88; *P* = 0.01). The LR+ of a ratio of 0.62 is 3.0 and the LR− is 0.42 (Table 2 and Figure 1).

## 4. Discussion 

In this study, the performance of the measurement of pNGAL and uNGAL for early detection of AKI is low, and the clinical usefulness could be questioned. 

Our study has several strengths. Firstly, we measured both plasma and urine NGAL concentration at the same time. Most of the previous studies were focused only on one setting [12–14]. Secondly, we used analysers from laboratory platforms allowing rapid information to the clinicians. This is known to improve accuracy and performance of NGAL in contrast to research-based assays (cutoff value >150 ng/mL) [7] most commonly used [10]. Thirdly, we took only one measure at 3 hours after cardiopulmonary bypass. This time corresponds to the peak of NGAL after a renal insult [12–15].

Lastly, the clinician in charge of the patient and the laboratory staff were respectively blinded from the results of the measurement and the clinical outcome.

However, this study has also some limitations, namely, the small number of patient and the single centre design. The measurement could also be performed later, but it will decrease the usefulness. 

There are some striking differences between our results and other studies concerning the ability of NGAL to predict AKI after cardiac surgery. In 2006, Wagener et al. found a sensibility and a specificity of 69% and 65% respectively, (AUC-ROC 0.73; 95%CI 0.504–0.97; *P* = 0.059) at 3 hours for uNGAL and 73% and 78% (AUC-ROC 0.8; 0.573–1.027; *P* = 0.017) at 18 hours. They concluded that uNGAL may be useful in prediction of AKI [12]. In 2009, Haase-Fielitz et al. found a sensibility and a specificity of 79% and 78% respectively for pNGAL on arrival in the ICU (AUC-ROC 0.80; 95%CI 0.63–0.96), and they concluded that pNGAL is superior to conventional biomarkers in prediction of AKI [14, 16].

Variations in the surgical and anaesthetic technique could unlikely explain these discrepancies as it is relatively standardized across different countries, and the AKI incidence is almost the same as previously reported according to the AKI definition [14, 16].

The first promising results for NGAL as a biomarker of AKI were obtained in paediatric cardiac surgery. NGAL in this setting showed an excellent sensitivity and specificity both on urinary [15] and plasmatic dosage [17] for early detection of patients at risk of developing an AKI (at 2 hours). These studies included a very standardized population suffering from isolated congenital heart disease and with little or no comorbidities such as latent chronic kidney disease [15, 17]. This could not be extrapolated to the heterogeneous population encountered in adult cardiac surgery. This is confirmed by the lowest performance of this biomarker in the studies in adult cardiac surgery [7, 18].

NGAL was studied in numerous others clinical settings with good results: contrast dye-induced nephropathy after percutaneous coronary procedures [19, 20] or use of contrast dye in children [21], critical illness in children [22, 23], or in adults [24] and in the emergency department [25]. However, the results of all these studies are difficult to extend because of important differences in AKI definitions, measurement methods, timing, population, and several possible confounding factors.

Due to its nature and intrinsic properties, NGAL is considered as a marker of tissue damage and tubular stress but not of kidney function or GFR like creatinine and urinary output. Actually, AKI in cardiac surgery patients is multifactorial including factors such as hypovolaemia, hypotension, chronic kidney disease, and drugs toxicity, that occurred at different timing before, during, and after the surgery. Therefore, isolated measurement of NGAL can not account for these mechanisms of AKI in this setting [26].

As focused by Honore et al., the sensitivity and specificity of individual biomarkers remain unacceptably low [27], and no single biomarker will be able to predict early AKI as well as AKI severity and duration in all clinical settings. 

Probably different set of biomarker at different time points could be more efficient to predict AKI according to the pathologies and mechanisms of AKI [26]. 

In a recent multicenter pooled analysis of prospective studies, Haase et al. pointed that in the absence of a diagnostic increase in serum creatinine, NGAL detects patients with likely subclinical AKI who have an increased risk of adverse outcomes and that the concept and definition of AKI might need reassessment [28].

Finally, technical problems may not be excluded. Indeed, Alere communicate that they will promptly launch a new version of the NGAL test on triage that should take in consideration low range values. In addition, concerning the cutoffs of uNGAL in healthy patients, there is some evidence of a large distribution of the 95th centile, with significantly influence of gender, age, and leukocyturia on this cut-offs value [29].

Thus, we need larger and quality-improved multicenter studies to define the exact areas of interest of all the new biomarkers and maybe technical improvement. 

## 5. Conclusions

The early detection of AKI remains one of the oldest challenges for intensivists, nephrologists, or every physician who is interested in the AKI management. This is the preliminary condition to a rapid initiation of a treatment and, therefore, a reduction in ICU stay, hospital stay, morbidity, and mortality. 

According to this study pNGAL, uNGAL, and uNGAL/ucreat ratio measurement 3 hours after cardiac surgery is not able to predict AKI with a sufficient clinical pertinence compared to the usual criteria at this time. Thus, this could not justify the cost of the analysis in routine.

## Figures and Tables

**Table 1:** uNGAL, pNGAL, and uNAGL/ucreatinine of the patients who developed AKI later according to the timing of creatinine rise.

Before cardiac surgery		After cardiac surgery				
GFR	pNGAL	AKI stage	pNGAL	uNGAL	uNGAL/ucreat.	
mL/min	ng/mL (>149 ng/mL)		ng/mL (>149 ng/mL)	ng/mL (>130 ng/mL)	ng/mg	ng/*μ*mol
	Creatinine rise 6 hours after cardiac surgery					

81	60	3	60	77	220	24.8
74	60	1	156	3.4	29	3.27
73	Not realised	1	177	Not realised		

	Creatinine rise ≤ 12 hours after cardiac surgery					

67	121	2	114	7.6	48	5.4
60	93	3	109	166	270	30.48
72	60	1	77	64.6	92	10.38
74	60	1	215	Not realised		
93	60	1	60	7.3	24	2.7
53	Not realised	1	212	117	500	56.4

	Creatinine rise ≥ 24 hours after cardiac surgery					

72	60	1	102	5.5	80	9.0
55	60	3	101	9.8	260	2.93
54	Not realised	1	75	11.2	79	8.92
61	Not realised	1	169	7.9	110	12.4
95	Not realised	2	163	28.4	189	21.3

	Dialysis					

29	60	3	301	4.2	63	7.11
40	Not realised	3	60	9.7	40	4.5
22	Not realised	3	161	37	120	13.5

**Table 2:** Area under the ROC curve for the pNGAL, uNGAL, and uNGAL/ucreatinine.

	pNGAL	uNGAL	uNGAL/ucreatinine
Area	0.5847	0.6250	0.7369
95% CI	0.415–0.753	0.440–0.809	0.587–0.886
*P* value	0.3307	0.1810	0.0112
Pts with no AKI	332	28	28
Pts with AKI	17	15	15

## References

[1] Uchino S, Kellum JA, Bellomo R (2005). Acute renal failure in critically ill patients: a multinational, multicenter study. *Journal of the American Medical Association*.

[2] Hoste EAJ, Kellum JA, Katz NM, Rosner MH, Haase M, Ronco C (2010). Epidemiology of acute kidney injury. *Contributions to Nephrology*.

[3] Stein A, de Souza LV, Belettini CR, Menegazzo WR, Viegas JR, Costa Pereira EM (2012). Fluid overload and changes in serum creatinine after cardiac surgery: predictors of mortality and longer intensive care stay. A prospective cohort study. *Critical Care*.

[4] Bagshaw SM, George C, Bellomo R (2008). A comparison of the RIFLE and AKIN criteria for acute kidney injury in critically ill patients. *Nephrology Dialysis Transplantation*.

[5] Mehta RL, Kellum JA, Shah SV (2007). Acute Kidney Injury Network: report of an initiative to improve outcomes in acute kidney injury. *Critical Care*.

[6] Bagshaw SM, Bellomo R (2007). Early diagnosis of acute kidney injury. *Current Opinion in Critical Care*.

[7] Moore E, Bellomo R, Nichol A (2010). Biomarkers of acute kidney injury in anesthesia, intensive care and major surgery: from the bench to clinical research to clinical practice. *Minerva Anestesiologica*.

[8] Mishra J, Qing MA, Prada A (2003). Identification of neutrophil gelatinase-associated lipocalin as a novel early urinary biomarker for ischemic renal injury. *Journal of the American Society of Nephrology*.

[9] Mishra J, Mori K, Ma Q, Kelly C, Barasch J, Devarajan P (2004). Neutrophil gelatinase-associated lipocalin: a novel early urinary biomarker for cisplatin nephrotoxicity. *American Journal of Nephrology*.

[10] Haase M, Bellomo R, Haase-Fielitz A (2010). Neutrophil gelatinase-associated lipocalin. *Current Opinion in Critical Care*.

[11] Levey AS, Stevens LA, Schmid CH (2009). A new equation to estimate glomerular filtration rate. *Annals of Internal Medicine*.

[12] Wagener G, Jan M, Kim M (2006). Association between increases in urinary neutrophil gelatinase-associated lipocalin and acute renal dysfunction after adult cardiac surgery. *Anesthesiology*.

[13] Wagener G, Gubitosa G, Wang S, Borregaard N, Kim M, Lee HT (2008). Urinary neutrophil gelatinase-associated lipocalin and acute kidney injury after cardiac surgery. *American Journal of Kidney Diseases*.

[14] Haase-Fielitz A, Bellomo R, Devarajan P (2009). Novel and conventional serum biomarkers predicting acute kidney injury in adult cardiac surgery—a prospective cohort study. *Critical Care Medicine*.

[15] Mishra J, Dent C, Tarabishi R (2005). Neutrophil gelatinase-associated lipocalin (NGAL) as a biomarker for acute renal injury after cardiac surgery. *The Lancet*.

[16] Haase M, Bellomo R, Devarajan P (2009). Novel biomarkers early predict the severity of acute kidney injury after cardiac surgery in adults. *Annals of Thoracic Surgery*.

[17] Dent CL, Ma Q, Dastrala S (2007). Plasma neutrophil gelatinase-associated lipocalin predicts acute kidney injury, morbidity and mortality after pediatric cardiac surgery: a prospective uncontrolled cohort study. *Critical Care*.

[18] Haase M, Bellomo R, Devarajan P, Schlattmann P, Haase-Fielitz A (2009). Accuracy of neutrophil gelatinase-associated lipocalin (NGAL) in diagnosis and prognosis in acute kidney injury: a systematic review and meta-analysis. *American Journal of Kidney Diseases*.

[19] Bachorzewska-Gajewska H, Malyszko J, Sitniewska E (2008). NGAL (neutrophil gelatinase-associated lipocalin) and cystatin C: are they good predictors of contrast nephropathy after percutaneous coronary interventions in patients with stable angina and normal serum creatinine?. *International Journal of Cardiology*.

[20] Ling W, Zhaohui N, Ben H (2008). Urinary IL-18 and NGAL as early predictive biomarkers in contrast-induced nephropathy after coronary angiography. *Nephron—Clinical Practice*.

[21] Hirsch R, Dent C, Pfriem H (2007). NGAL is an early predictive biomarker of contrast-induced nephropathy in children. *Pediatric Nephrology*.

[22] Zappitelli M, Washburn KK, Arikan AA (2007). Urine neutrophil gelatinase-associated lipocalin is an early marker of acute kidney injury in critically ill children: a prospective cohort study. *Critical Care*.

[23] Wheeler DS, Devarajan P, Ma Q (2008). Serum neutrophil gelatinase-associated lipocalin (NGAL) as a marker of acute kidney injury in critically ill children with septic shock. *Critical Care Medicine*.

[24] Siew ED, Ware LB, Gebretsadik T (2009). Urine neutrophil gelatinase-associated lipocalin moderately predicts acute kidney injury in critically ill adults. *Journal of the American Society of Nephrology*.

[25] Nickolas TL, O’Rourke MJ, Yang J (2008). Sensitivity and specificity of a single emergency department measurement of urinary neutrophil gelatinase-associated lipocalin for diagnosing acute kidney injury. *Annals of Internal Medicine*.

[26] Koyner JL (2012). Assessment and diagnosis of renal dysfunction in the ICU. *Chest*.

[27] Honore PM, Jacobs R, Joannes-Boyau O, Verfaillie L, De Regt J, Van Gorp V (2012). Biomarkers for early diagnosis of AKI in the ICU: ready for prime time use at the bedside?. *Annals of Intensive Care*.

[28] Haase M, Devarajan P, Haase-Fielitz A (2011). The outcome of neutrophil gelatinase-associated lipocalin-positive subclinical acute kidney injury: a multicenter pooled analysis of prospective studies. *Journal of the American College of Cardiology*.

[29] Cullen MR, Murray PT, Fitzgibbon MC (2012). Establishment of a reference interval for urinary neutrophil gelatinase-associated lipocalin. *Annals of Clinical Biochemistry*.

